# Preparation of Ethylene Glycol Dimethacrylate (EGDMA)-Based Terpolymer as Potential Sorbents for Pharmaceuticals Adsorption

**DOI:** 10.3390/polym12020423

**Published:** 2020-02-12

**Authors:** Nur Syafiqah Shaipulizan, Siti Nurul Ain Md Jamil, Sazlinda Kamaruzaman, Nur Nida Syamimi Subri, Abel Adekanmi Adeyi, Abdul Halim Abdullah, Luqman Chuah Abdullah

**Affiliations:** 1 Department of Chemistry, Faculty of Science, Universiti Putra Malaysia, UPM Serdang 43400, Selangor, Malaysia; syafiqahshaipulizan@gmail.com (N.S.S.); sazlinda@upm.edu.my (S.K.); nurnidasyamimi@gmail.com (N.N.S.S.); halim@upm.edu.my (A.H.A.); 2 Centre of Foundation Studies for Agricultural Science, Universiti Putra Malaysia, UPM Serdang 43400, Selangor, Malaysia; 3 Department of Chemical and Environmental Engineering, Faculty of Engineering, Universiti Putra Malaysia, UPM Serdang 43400, Selangor, Malaysia; abeladeyi@abuad.edu.ng (A.A.A.); chuah@upm.edu.my (L.C.A.); 4 Department of Chemical and Petroleum Engineering, College of Engineering, Afe Babalola University Ado-Ekiti, ABUAD, KM. 8.5, Afe Babalola Way, P.M.B. 5454, Ado-Ekiti 360211, Nigeria; 5 Institute of Advanced Technology, Universiti Putra Malaysia, UPM Serdang 43400, Selangor, Malaysia

**Keywords:** hypercrosslinked polymer, polyacrylonitrile, polar functional group, precipitation polymerization, batch adsorption, salicylic acid, mefenamic acid

## Abstract

Ethylene glycol dimethacrylate (EGDMA) is used as a crosslinker in poly(acrylonitrile (AN)-*co*-vinylbenzyl chloride (VBC)) to investigate the effect of long-chain crosslinker to the porosity of the terpolymer system. Poly(AN-*co*-EGDMA-*co*-VBC) is synthesized by using precipitation polymerization method and further hypercrosslinked by Friedel-Crafts reaction. FT-IR spectra of poly(AN-*co*-EGDMA-*co*-VBC) show that the absorption bands at ~1290 cm^−1^ that are assigned to the C–Cl vibrations are almost disappeared in hypercrosslinked (HXL) poly(AN-*co*-EGDMA-*co*-VBC) polymers, confirming that the hypercrosslinking reaction is successful. SEM images show that the morphologies of the polymers are retained through the hypercrosslinking reactions. Brunauer–Emmett–Teller (BET) analysis shows that hypercrosslinked polymers had a specific surface area up to 59 m^2^·g^−1^. The preliminary performance of the terpolymer adsorbent to capture polar analyte is evaluated by adsorbing salicylic acid and mefenamic acid from aqueous solution in a batch system. The maximum adsorption capacity of salicylic acid and mefenamic acid were up to 416.7 mg·g^−1^ and 625 mg·g^−1^, respectively, and the adsorption kinetic data obeyed pseudo-second-order rate equation.

## 1. Introduction

The presence of pharmaceuticals in water resources has raised concerns among researchers worldwide due to their ecotoxicological effects on human and aquatic life [1]. Pharmaceuticals are usually present in water at concentrations in the µg·L^−1^ to ng·L^−1^ range and this range is considered to be a potential threat to the environment [2,3]. Salicylic acid (the main metabolite of aspirin) and mefenamic acid which are classified as non-steroidal anti-inflammatory drugs (NSAIDs) have anti-inflammatory, analgesic, and antipyretic activity [4]. Low biodegradability and frequent use of these drugs caused them to be found in the Langat River samples at 475.2 ng·L^−1^ and 595.2 ng·L^−1^ maximum concentrations, respectively [5].

Adsorption is the most effective treatment technology for the removal of pharmaceuticals due to its simplicity, low cost and adaptability to a broad range of pharmaceuticals. Several adsorbents have been synthesized and analyzed their adsorption application mainly for pharmaceuticals. However, poor selectivity, low surface polarity, long contact time, and low adsorption capacity of adsorbents are limitation to the adsorption application. For instance, adsorption of salicylic acid using magnetic molecularly imprinted polymer reported by Zhang et al. [6] had a maximum adsorption capacity up to 36.8 mg·g^−1^. Adsorption of mefenamic acid and ibuprofen reported by Khalaf et al. [7] using clay micelle complex was up to 100 mg·g^−1^ of and adsorption using clay as an adsorbent was 90.9 mg·g^−1^ of maximum adsorption capacity. Poly allyl glycidyl ether/iminodiacetic acid-*co*-*N*,*N*-dimethylacrylamide grafted to silica gel prepared by Sadeghi and his group were utilized as adsorbents for the removal of mefenamic acid and the maximum adsorption capacity measured was only 7.0 mg·g^−1^ [8].

In recent years, hypercrosslinked polymers have been attracting increasing attention in the field of adsorption due to their high Brunauer–Emmett–Teller (BET) surface area (1000 m^2^·g^−1^) and a large number of micropores [9,10,11,12,13,14,15,16,17,18,19,20,21,22]. It has been reported that high maximum adsorption capacity of salicylic acid was obtained (up to 621 mg·g^−1^) by utilizing the hypercrosslinked resins as the adsorbents [15,19,20]. In addition, high adsorption capacity of diclofenac (up to 383.6 mg·g^−1^) using hypercrosslinked poly(acrylonitrile (AN)-*co*-divinylbenzene (DVB)-*co*-vinylbenzyl chloride (VBC)) as adsorbents to remove diclofenac was reported by Subri et al. [14]. Yu and his team reported the adsorption of p-aminobenzoic acid (pharmaceutical intermediates) by modified hypercrosslinked chloromethylated styrene/divinylbenzene with the maximum adsorption capacity of 300 mg·g^−1^ [23]. Zhang et al. [16] and Wang et al. [18] introduced ethylene glycol dimethacrylate (EGDMA) as a polar crosslinking agent and vinylbenzyl chloride (VBC), where the chloromethyl (-CH_2_Cl) substituent acts as an internal electrophile in preparing hypercrosslinked polymers. The synthesis of hypercrosslinked polymers by Friedel–Crafts reactions involved the conversion of chloromethyl group into methylene bridge where chlorine atom utilized as a leaving group to form methylene bridges between adjacent phenyl rings and therefore, lead to the formation of abundant pores inside the precursor polymer [24,25,26,27,28,29]. It was reported that incorporation of 90% of VBC in the feed (in poly(EGDMA-*co*-VBC) system) resulted in the formation of hypercrosslinked particles with high porosity; 1066 m^2^·g^−1^ [18] and 827 m^2^·g^−1^ [16]. Hypercrosslinked polymer composed of acrylonitrile (AN) as the polar monomer, divinylbenzene (DVB) as a crosslinker and VBC was reported by Subri et al. [14]. AN was incorporated into the terpolymer system to exploit the presence of nitrile group along the polymer chains [14]. Subri and coworkers reported that the hypercrosslinked (HXL) poly(AN-*co*-DVB-*co*-VBC) had a high specific surface area up to 1078 m^2^·g^−1^ [14]. Polymeric materials typically crosslinked with DVB produced a hydrophobic backbone where interactions with analyte molecules are strictly through π–π interactions [30]. DVB is known as a rigid crosslinker due to its aromatic group that prevents the packing of polymeric chains [31]. Introduction of long, flexible EGDMA into the polymer system provides greater flexibility and spacing between the polymer chains which favor the accessibility and attachments of analyte molecules to the functional groups of EGDMA [32]. In addition, the hydrophilic and polar character of EGDMA allows a high affinity to the aqueous phase and does not exhibit steric constraints when it is used as an adsorbent [31].

In the present work, AN was introduced as the polar monomer into a terpolymer system to increase the adsorbent surface polarity. VBC was used as a monomer to exploit the methyl chloride groups during hypercrosslinking via Friedel–Crafts reaction. To the best of our knowledge, the utilization of EGDMA as a crosslinking agent in AN-VBC copolymer system has never been reported before. It was widely reported that the most common crosslinkers that have been used in the polymeric system are DVB-80 [14,17,19,33] and *N*,*N*-methylenebisacrylamide [34,35,36,37]. EGDMA is a hydrophilic crosslinking agent with more polar characteristic compared to the DVB-80. Thus, it is interesting to investigate the effect of using EGDMA in the AN-VBC system. The effect of the feeding amount of the monomers (AN and EGDMA) was studied to investigate the influence of respective monomers towards the porosity of terpolymers. The potential of the hypercrosslinked terpolymers to capture polar molecules were preliminarily studied (in batch adsorption system) by using salicylic acid and mefenamic acid as polar analytes.

## 2. Experimental

### 2.1. Materials

Acrylonitrile (AN) was supplied by Merck (Darmstadt, Germany). Ethylene glycol dimethacrylate (EGDMA) and vinylbenzyl chloride (VBC) were purchased from Sigma-Aldrich (Dorset, UK). The initiator, benzoyl peroxide (BPO), was supplied by Sigma-Aldrich (Dorset, UK) and purified by recrystallization using acetone (Systerm ChemAR, Shah Alam, Malaysia) as the solvent at low temperature. Acetonitrile (HPLC grade), toluene, methanol, nitrobenzene, salicylic acid, and mefenamic acid were supplied by Sigma-Aldrich (Dorset, UK). AN, EGDMA, and VBC were purified by passing them through a short column of neutral alumina (Merck, Darmstadt, Germany) to remove inhibitors before polymerization. All other reagents were used as received.

### 2.2. Synthesis of Poly(AN-co-EGDMA-co-VBC)

**Figure 1:**
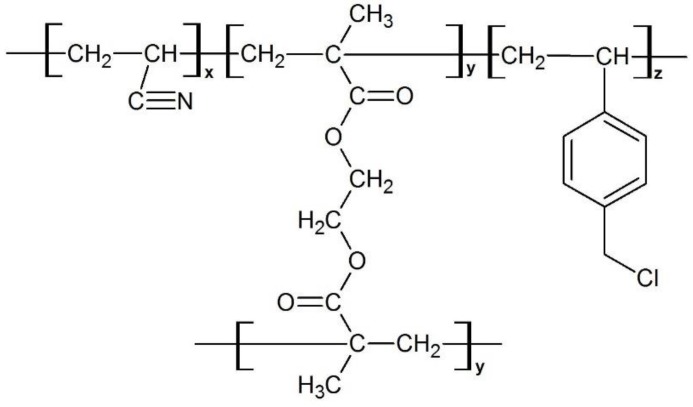
Theoretical chemical structure of poly(acrylonitrile (AN)-*co*-ethylene glycol dimethacrylate (EGDMA)-*co*-vinylbenzyl chloride (VBC)).

Polymers were synthesized by precipitation polymerization according to the previously reported method [14]. Figure 1 depicts the incorporation of AN into EGDMA and VBC as terpolymers. The specific feeding amount of monomers is tabulated in Table 1. The inclusion of 5 mol% of VBC in the terpolymer system is due to the reference by Subri et al. [14] which reported that the inclusion of 5 mol% of VBC into the AN/DVB-80 system was sufficient to enhance the specific surface area up to 1078 m^2^·g^−1^. Thus, instead of using DVB as a crosslinking monomer, this work is to study the incorporation of a different type of crosslinking monomer, which is EGDMA, in the terpolymer system, due to its larger molecular weight and molecular length compared to the DVB-80. The monomers with a total concentration of 2% (*w/v*) and initiator (BPO) with a concentration of 2% (*w/w*) were dissolved in a mixture of acetonitrile (150 mL) and toluene (50 mL) in a polypropylene Nalgene bottle fitted with a screw cap. Acetonitrile and toluene acted as porogens in this reaction. The bottle was sealed with parafilm and placed in a shaker bath for 30 min. The monomer solution in the bottle was deoxygenated by sparging with N_2_ in an ice bath for 30 min. The bottle was resealed and placed on a low profile roller and rotated slowly (30 rpm) in a Stuart Scientific S160 incubator. The temperature of the incubator was increased from ambient to 60 °C for 2 h and the polymerization was allowed to proceed for 96 h. The formed polymer particles were filtered on a 0.2 µm nylon membrane filter and then washed in sequence with acetonitrile, toluene, methanol, and acetone. The polymer sample was dried in a vacuum oven overnight at 40 °C.

### 2.3. Synthesis of Hypercrosslinked Poly(AN-co-EGDMA-co-VBC)

**Figure 2:**
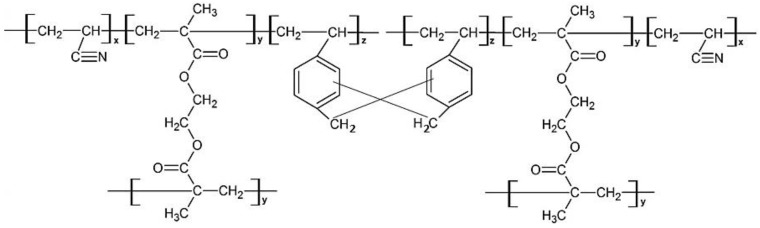
Theoretical chemical structure of hypercrosslinked (HXL) poly(AN-*co*-EGDMA-*co*-VBC).

A total of 1.0 g of poly(AN-*co*-EGDMA-*co*-VBC) was added to 30 mL of nitrobenzene in a 100 mL three-neck round-bottomed flask. The solution mixture was then purged with N_2_ at room temperature for 1 h to promote the hypercrosslinking reaction. Iron (III) chloride (FeCl_3_) in nitrobenzene (20 mL) was added and the mixture was heated at 80 °C for 18 h. The polymer particles were filtered and then extracted overnight with acetone in a Soxhlet extractor. The polymer particles were washed again with methanol before drying in a vacuum oven at 40 °C. The proposed chemical structure of poly(AN-*co*-EGDMA-*co*-VBC) after the hypercrosslinking reaction is shown in Figure 2.

### 2.4. Batch Adsorption Study

The adsorption capacity of HXL poly(AN-*co*-EGDMA-*co*-VBC) for the removal of salicylic acid and mefenamic acid was studied in a batch system. The batch experiment was performed using 5 mg of HXL poly(AN-*co*-EGDMA-*co*-VBC) mixed with 10 mL of pharmaceutical solutions at 100–500 mg·L^−1^ of initial concentrations. The mixture was shaken at 150 rpm in a temperature-controlled water bath shaker (Model-903, Tech-Lab Mfg Sdn Bhd, Malaysia) at room temperature for 4 h. Then 3 mL aliquot of the supernatant were taken out and analyzed by Lambda 35 UV-Vis spectrometer (Perkin Elmer Life and Analytical Science, Singapore) at 296 nm (salicylic acid) and 285 nm (mefenamic acid) maximum wavelength. The experiments were performed in triplicate and the average values were recorded.

The residual concentration of the adsorbate, *C_e_* (mg·L^−1^) was determined and the equilibrium adsorption capacity, *q_e_* (mg·g^−1^) was calculated as:

(1) $$q_{e}=\frac{(C_{o}-C_{e})V}{m}$$

where *C_o_* is the initial concentration of the adsorbate (mg·L^−1^), *V* is the volume of the solution (L), and *m* is the amount of adsorbent used (g).

Kinetic studies were carried out in a temperature-controlled water bath shaker (Model-903, Tech-Lab Mfg Sdn Bhd, Malaysia) at 150 rpm, using 100 mg·L^−1^ of the initial concentration of pharmaceutical solution and known HXL poly(AN-*co*-EGDMA-*co*-VBC) dose. The samples were withdrawn at set time intervals (1–25 min), filtered, and supernatant pharmaceutical solution concentration was analyzed. The equilibrium and kinetic studies of the adsorption of salicylic acid and mefenamic acid by poly(AN-*co*-EGDMA-*co*-VBC) and poly(AN) were used as controls.

### 2.5. Characterizations

Fourier Transform Infrared (FT-IR) spectra of the samples was recorded using a Spectrum 100 Perkin Elmer (Waltham, MA, USA) with the wavenumber range from 280 to 4000 cm^−1^. Carbon, hydrogen, and nitrogen contents of the polymers were measured by carbon hydrogen nitrogen sulphur (CHNS) analyzer (CHNS-932 Leco Corporation, St. Joseph, MI, USA). The morphology, oxygen, and chlorine contents of the polymer particles was observed using Scanning Electron Microscopy-Energy Dispersive X-ray (SEM-EDX) (JEOL JSM 6360LA, Tokyo, Japan). The pore structure was determined by N_2_ adsorption-desorption isotherms via a Micromeritics Instrument Corporation, Model-3Flex (Norcross, GA, USA) surface area and porosity analyzer. Samples were degassed overnight under vacuum at 100 °C and then analyzed using nitrogen sorption that was carried out at 77 K. The BET surface area and pore volume were calculated according to the BET model.

## 3. Results and Discussion

### 3.1. Yield of Polymerization

Table 2 shows the yields of polymerization of poly(AN) and poly(AN-*co*-EGDMA-*co*-VBC) that were isolated after 96 h of reaction time. The yield of poly(AN) is low at 3% due to its high solubility in the porogen (acetonitrile and toluene). Thus, the recovery process involved evaporation of solvent overnight and decanting the solvent to separate particles from the mixture. This process might have caused low yield after the recovery process. The yield of poly(AN-*co*-EGDMA-*co*-VBC) 25/70/5 was 39% while the yield of poly(AN-*co*-EGDMA-*co*-VBC) 20/75/5 was 48%. The lower yield of poly(AN-*co*-EGDMA-*co*-VBC) 25/70/5 compared to poly(AN-*co*-EGDMA-*co*-VBC) 20/75/5 might be due to the higher amount of AN introduced into the terpolymer system. The formation of precipitates in the middle of reaction indicates the polymer formation, but at the same time, increasing of AN concentration preventing the incorporation of more monomer units due to the difficulty of AN incorporation to polymer chains during chains growing [31]. Faster incorporation of EGDMA crosslinker to the growing polymer chains compared to AN also contributed to the high yield of polymerization. This is because the methacrylate groups from EGDMA exhibited higher reactivity than the acrylate groups from the AN monomer [31]. Meanwhile, low yields of homopolymer (poly(AN)) (3%) might be due to the absence of crosslinking monomer that usually stabilized the particles in the continuous phase and prevents precipitation at an early stage [38]. This also indicates that the incorporation of AN is difficult within the polymer. The observation from the yield of polymerization shows that the presence of EGDMA helps in stabilizing the chain growth during polymerization that results in higher yield.

### 3.2. Physiochemical Characterization

**Figure 3:**
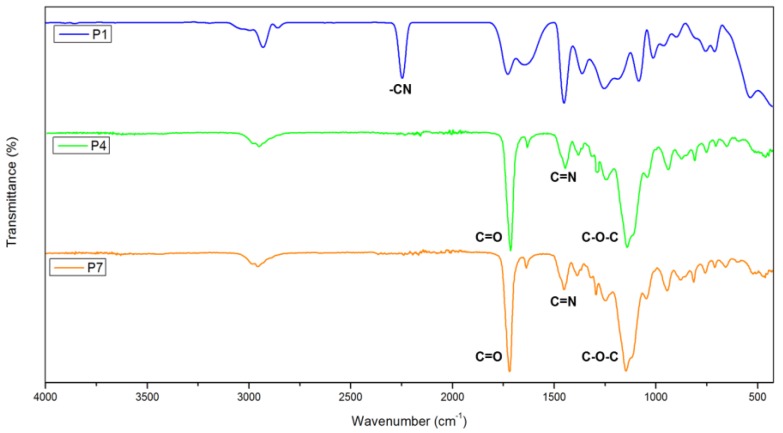
FT-IR spectra of poly(AN) (P1), poly(AN-*co*-EGDMA-*co*-VBC) 25/70/5 (P4), and poly(AN-*co*-EGDMA-*co*-VBC) 20/75/5 (P7).

Figure 3 shows the FT-IR spectra of poly(AN), poly(AN-*co*-EGDMA-*co*-VBC) 25/70/5 (P4), and poly(AN-*co*-EGDMA-*co*-VBC) 20/75/5 (P7). The FT-IR spectrum of poly(AN) shows an absorption band at 2243 cm^−1^ that corresponds to the stretching of the nitrile group (−C≡N), C-H stretching at 2935 cm^−1^ and bending vibration of –CH_2_ group at 1452 cm^−1^. From the FT-IR spectra of P4 and P7, the absorption band identified at 2960 cm^−1^ is due to the CH_3_ vibration [31]. The strong vibration at 1720 cm^−1^ corresponds to the ester carbonyl groups of EGDMA [39], and the absorption band at 1147 cm^−1^ is due to the C–O–C stretching vibration [31]. The absorption band that was assigned to the C≡N stretching vibration was not present in the spectra of P4 and P7. However, C=N stretching was identified at 1450 cm^−1^ [14]. This is due to the presence of an active radical initiator (BPO) which contain oxygen atoms that initiated the hydrolysis of −C≡N into C=N groups during the polymerization reaction [14,40]. The C–Cl stretching of chloromethyl groups at 1290 cm^−1^ [41] confirms the incorporation of VBC monomer into the polymer chains. 

**Figure 4:**
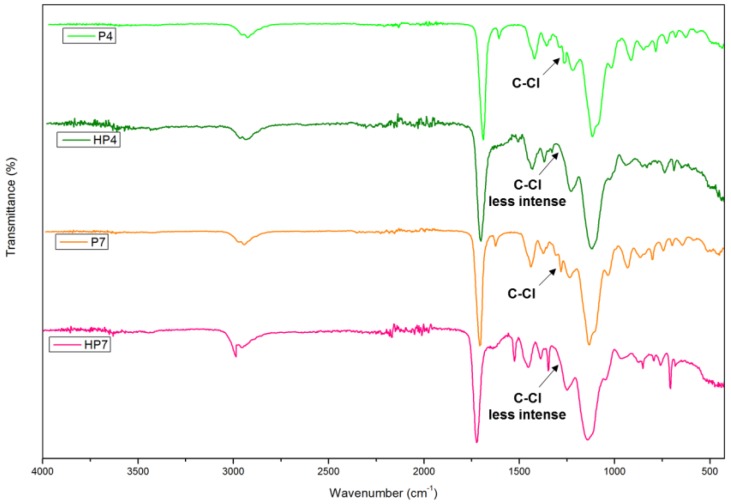
FT-IR spectra of poly(AN-*co*-EGDMA-*co*-VBC) before (P4, P7) and after (HP4, HP7) the hypercrosslinking reaction.

**Figure 5:**
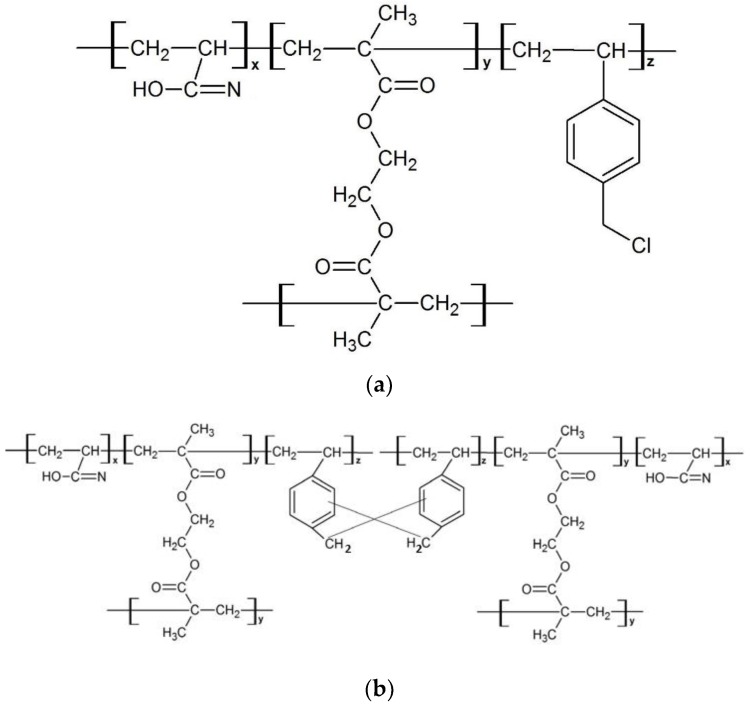
Chemical structure of (**a**) poly(AN-*co*-EGDMA-*co*-VBC) (P4, P7) and (**b**) HXL poly(AN-*co*-EGDMA-*co*-VBC) (HP4, HP7).

Figure 4 displays the FT-IR spectra of poly(AN-*co*-EGDMA-*co*-VBC) before and after the hypercrosslinking reaction. The C–Cl stretching of chloromethyl groups at 1290 cm^−1^ almost disappeared after hypercrosslinking reaction indicate that the chloromethyl groups were consumed through hypercrosslinking [14] and the rigid methylene cross-linking bridges were connected in the polymer chains [17]. Figure 5 presented the proposed chemical structure of poly(AN-*co*-EGDMA-*co*-VBC) and HXL poly(AN-*co*-EGDMA-*co*-VBC) based on the FT-IR spectra displayed in Figure 3 and Figure 4.

CHNS analysis was used to estimate the composition of carbon, hydrogen and nitrogen in the polymers. Meanwhile, oxygen and chlorine contents of the polymers were determined using EDX analysis. Table 3 shows the C, H, N, O, and Cl contents of poly(AN), and poly(AN-*co*-EGDMA-*co*-VBC) before and after hypercrosslinking reactions. Sample P1 contains the highest N contents since there are no comonomers involved. In the case of P4 and P7, the N content decreased from 0.51% to 0.40% and O content increased from 27.40% to 29.81%, which is in agreement with their AN and EGDMA composition in the monomer feed. Based on the EDX analysis data shown in Table 3, the chlorine content of poly(AN-*co*-EGDMA-*co*-VBC) (P4) decreased from 7.55% to 5.48% after hypercrosslinking reaction, indicate that chloromethyl groups were being consumed during hypercrosslinking reaction. Thus, this indicate that hypercrosslinking of poly(AN-*co*-EGDMA-*co*-VBC) was successfully carried out.

**Figure 6:**
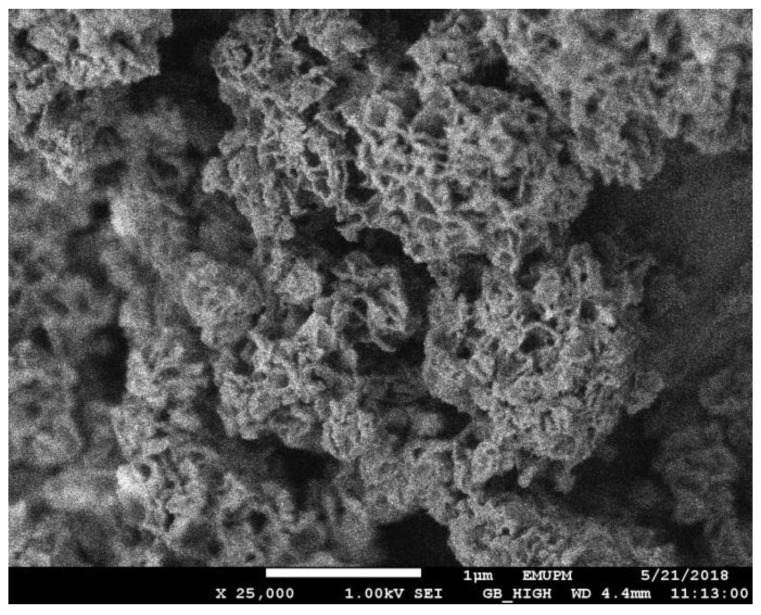
Scanning electron micrograph of poly(AN) (P1).

**Figure 7:**
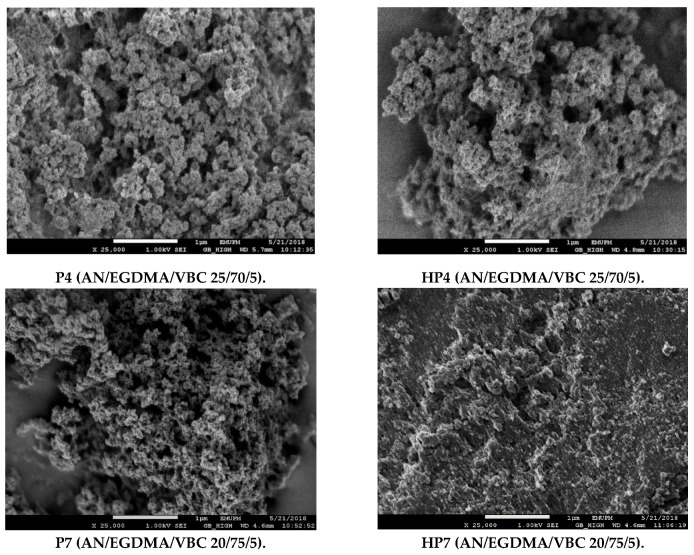
Scanning electron micrographs of poly(AN-*co*-EGDMA-*co*-VBC) before (**left**) and after (**right**) hypercrosslinking reaction.

Figure 6 and Figure 7 presented the scanning electron micrographs of poly(AN) and poly(AN-*co*-EGDMA-*co*-VBC) before and after the hypercrosslinking reaction, respectively. Poly(AN) appears as agglomerated clusters of small particles. The morphologies of P4, HP4, P7 and HP7 had agglomerated features. Galicia-Aguilar and coworkers reported the same morphologies of the synthesized AN/EGDMA polymeric adsorbent which showed a sponge-like appearance and agglomerated cluster of particles [31]. The morphologies of the polymers (HP4 and HP7) were successfully retained even when the high temperature (100 °C) was used. The ability of the hypercrosslinked polymer to retain their initial shape even after 18 h of reaction time might be due to the abundance amount of EGDMA crosslinker used during precursor polymer synthesis that provides network stability in the terpolymer system. EGDMA crosslinker prevents the polymer chains from collapsing in the swollen state by holding the polymer chains together [24].

As presented in Table 4, the specific surface area of P7 and P4 with 20 and 25 mol% of AN in the monomer feed were 47.4 m^2^·g^−1^ and 45.6 m^2^·g^−1^, respectively. Low specific surface area of the polymer might due to the high amount of EGDMA introduced into the polymeric system. In addition, low surface area of poly(AN-EGDMA) (61–298 m^2^·g^−1^) was also reported by Galicia-Aguilar et al. [31] and this is due to the presence of AN that caused a higher degree of aggregation between particle which then caused a decreasing in the specific surface area of the polymer. After the hypercrosslinking reaction, the specific surface area of P4 was slightly increased from 45.6 to 59.1 m^2^·g^−1^. However, the surface area of the polymer is not as high as reported by Subri et al. [14] which used divinylbenzene-80 (DVB-80) as a crosslinking agent in the poly(AN-*co*-DVB-80-*co*-VBC) system. Polymers synthesized by Subri et al. [14] in a mixture of acetonitrile and toluene as the porogen had a high specific surface area up to 1078 m^2^·g^−1^. Fu and his team reported high specific surface area (1047 m^2^·g^−1^) of poly(methyl acrylate (MA)-*co*-DVB-*co*-VBC) synthesized in toluene as a porogen [19]. This indicates that toluene and acetonitrile are good porogens as well as a precipitating agent for polymers based upon DVB. The use of suitable and good porogens give rapid phase separation during the process of polymerization which caused the polymer to precipitate more quickly and hence give porous structure in the polymer microspheres which results in high number of pore and higher specific surface area [42]. As reported by Li et al. [43], polymerization of EGDMA in acetonitrile resulted in the formation of non-spherical particles. The low specific surface area of hypercrosslinked polymer (16.8 m^2^·g^−1^) using EGDMA as a crosslinker, 5 mol% of VBC, and toluene as porogen was also reported by Yahya and Abdullah [24]. Meanwhile, in the present work, the specific surface area of P7 showed a decrease in specific surface area which is from 47.4 to 1.1 m^2^·g^−1^ after the hypercrosslinking reaction. Hence, in the case of porosity analysis, we may conclude that low specific surface area of HP7 was due to the chemical reactions during hypercrosslinking that disrupted the wall of the pores and thus resulted to high mean pore size (447 nm).

The presence of a stabilizer such as polyvinyl alcohol (PVA) or poly(*N*-vinylpyrrolidone) (PVP) in synthesizing EGDMA-based polymer plays a big role in producing well-defined spherical particles (beads) [24,44] with high porosity polymer microspheres [16,18]. The stabilizers hinder the coalescence of monomer droplets and then stabilize the polymer beads that have a tendency to agglomerate [45]. Low porosity also might be due to the packing of the polymeric chain caused by the presence of EGDMA with flexible chain. The low percentage of VBC in the terpolymer system is also one of the factors that cause the specific surface area to be lower than expected [20,46]. A high percentage of VBC caused high residual chlorine content in the products, hence the amount of methylene bridging is also high [24]. Hence, low specific area of HXL poly(AN-*co*-EGDMA-*co*-VBC) (59.1 m^2^·g^−1^) is due to the plenty formation of methylene bridges between adjacent phenyl rings and therefore, lead to the low formation of pores inside the polymer.

### 3.3. Adsorption Study

#### 3.3.1. Effect of Initial Concentration

**Figure 8:**
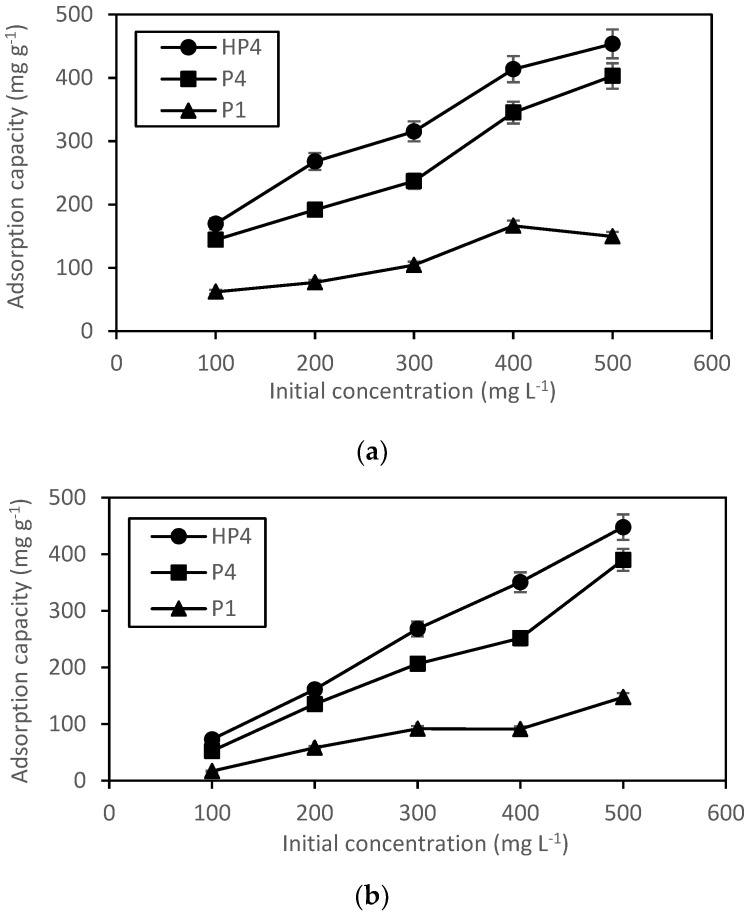
Adsorption capacity of (**a**) salicylic acid and (**b**) mefenamic acid on HP4, P4, and P1. (Error bars represent the standard deviation of triplicate recordings). Condition: 5 mg of adsorbent in 10 mL of 100–500 mg·L^−1^ of pharmaceuticals solution at 150 rpm for 4 h at room temperature of 25 °C.

Figure 8 represents the effect of the initial concentration of pharmaceuticals using HXL poly(AN-*co*-EGDMA-*co*-VBC) (HP4), poly(AN-*co*-EGDMA-*co*-VBC) (P4), and poly(AN) (P1) as adsorbents. The adsorption capacity of pharmaceuticals (salicylic acid and mefenamic acid) by HXL poly(AN-*co*-EGDMA-*co*-VBC), poly(AN-*co*-EGDMA-*co*-VBC), and poly(AN) at various concentrations increase as the concentration of pharmaceuticals is increased. The adsorption capacity of pharmaceuticals on HXL poly(AN-*co*-EGDMA-*co*-VBC) is higher than that of poly(AN-*co*-EGDMA-*co*-VBC) and poly(AN), which may be due to the increased surface area after Friedel–Crafts reaction of poly(AN-*co*-EGDMA-*co*-VBC). Figure 8a,b shows that salicylic acid and mefenamic acid uptake by HXL poly(AN-*co*-EGDMA-*co*-VBC) increased from 169.5 to 453.6 mg·g^−1^ and 73.1 to 447.8 mg·g^−1^, respectively.

#### 3.3.2. Adsorption Isotherm

The equilibrium adsorption isotherm is important in explaining the interactive behavior between the adsorbate and adsorbent. The parameters of Langmuir and Freundlich equations such as *q_m_*, *K_L_*, *K_F_*, and n as well as the correlation coefficients (R^2^) are summarized in Table 5 (Figures S1 and S2 present the graph of Langmuir and Freundlich isotherms, respectively). The results from the correlation coefficient of the adsorption isotherm showed that the equilibrium data of salicylic acid can be well fitted with Freundlich model, assuming that the adsorption of adsorbents towards salicylic acid was multilayer adsorption and the adsorption occurred at sites with different energy of adsorption [18]. Additionally, the value of n ˃ 1 indicates favorable process and strong bond exist between salicylic acid and adsorbents [18,47,48]. The greatest value of *K_F_* for HXL poly(AN-*co*-EGDMA-*co*-VBC) (HP4) among these polymers suggested the most efficient adsorption of salicylic acid. In the case of mefenamic acid, good correlation coefficient of the Langmuir model suggested that the experimental data are in better agreement with the Langmuir model implying monolayer adsorption onto an adsorbent surface [18,49], where one molecule can be adsorbed on one active site [49]. The linear Langmuir [20] and Freundlich [50,51] isotherm are described by the following equations, respectively:

(2) $$\frac{1}{q_{e}}=\;\frac{1}{K_{L}\;q_{m}}\cdot \frac{1}{C_{e}}\;+\;\frac{1}{q_{m}}$$

(3) $$\ln\;q_{e}\;=\;\frac{1}{n}\;\ln\;C_{e}\;+\;\ln\;K_{F}$$

where *q_m_* is the maximum adsorption capacity on the adsorbent (mg·g^−1^), *K_L_* is the Langmuir constant (L·mg^−1^), *K_F_* ((mg/g) (L/mg)1/n) and n are the Freundlich constants.

The maximum adsorption capacity of salicylic acid and mefenamic acid predicted by Langmuir model were up to 416.7 mg·g^−1^ and 625 mg·g^−1^, which are larger than some other reported adsorbents (Table 6) [6,7,8,52]. The adsorption capacity measured is also comparable to other reported adsorbents (Table 6) [15,19] despite of having low specific surface area. The active functional group contributed by imine group derived from the nitrile group of AN, carbonyl group from EGDMA, and the long, flexible chain of EGDMA give high adsorption capacity towards polar analytes even though the specific surface area of the polymer is low.

#### 3.3.3. Effect of Contact Time

**Figure 9:**
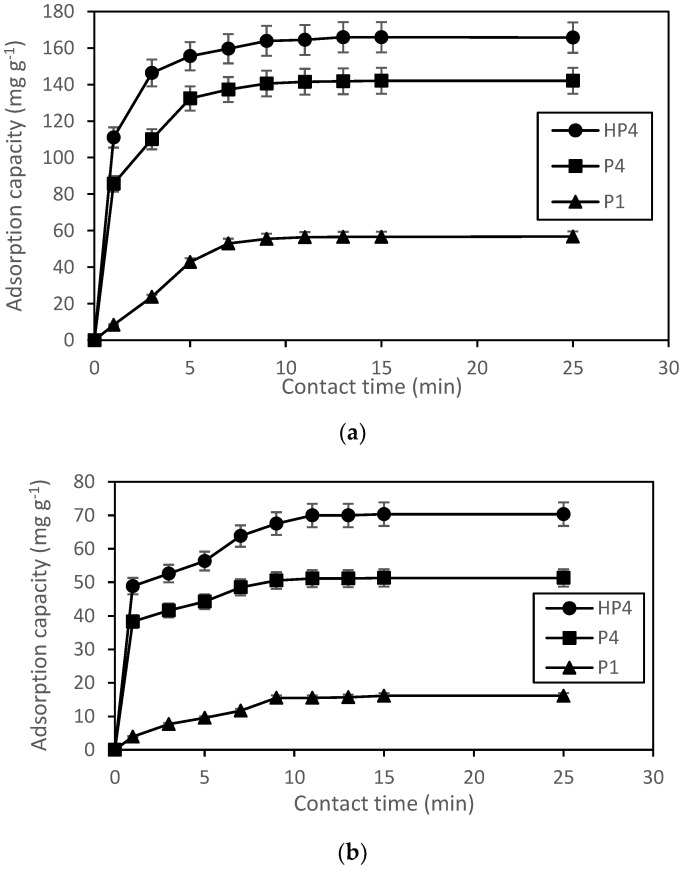
Adsorption capacity of (**a**) salicylic acid and (**b**) mefenamic acid on HP4, P4, and P1. (Error bars represent the standard deviation of triplicate recordings). Condition: 5 mg of adsorbent in 10 mL of 100 mg·L^−1^ of pharmaceuticals solution (10 mg of pharmaceuticals in 100 mL of methanol) at 150 rpm at room temperature of 25 °C.

Adsorption capacity was measured as a function of contact time to determine the optimum contact time for the adsorption of pharmaceuticals (salicylic acid and mefenamic acid) on HP4, P4, and P1. As shown in Figure 9a,b, the adsorption capacity increased as the contact time increased until the optimum contact time was reached at a certain time, and the adsorption reached equilibrium within 15 min, indicating fast adsorption process. For salicylic acid, HP4 and P4 reached optimum contact time of 13 min with 165.9 mg·g^−1^ and 142.0 mg·g^−1^ of adsorption capacity, respectively. In contrast, P1 reached optimum contact time of 11 min with 56.4 mg·g^−1^ adsorption capacity. Meanwhile, for mefenamic acid, HP4 and P4 reached optimum contact time of 11 min with 70.3 mg·g^−1^ and 51.3 mg·g^−1^ of adsorption capacity, respectively. In contrast, P1 reached optimum contact time of 15 min with 16.1 mg·g^−1^ adsorption capacity. HXL poly(AN-*co*-EGDMA-*co*-VBC) has the highest adsorption capacity compared to poly(AN-*co*-EGDMA-*co*-VBC) and poly(AN) due to its high number of available active sites. The equilibrium point is reached might probably because of the active site of the polymers was fully occupied with polar analytes [53]. Therefore, there were no available sites on the polymer particles to adsorb more polar analytes. By comparing the results in this study to the other studies in Ref. [15] (hypercrosslinked DVB/VBC, *t* = 90 min), Ref. [15] (phenoxy modified hypercrosslinked DVB/VBC, *t* = 55 min), Ref. [19] (hypercrosslinked poly(VBC-*co*-DVB-*co*-MA), *t* = 60 min), Ref. [6] (magnetic molecularly imprinted polymer, *t* = 30 min), and Ref. [52] (Poly urea formaldehyde-bentonite, *t* = 45 min), the polymers in this study had a much faster adsorption rate.

#### 3.3.4. Adsorption Kinetics

The kinetic data were analyzed using the pseudo-first-order and pseudo-second-order models. The linearized form of pseudo-first-order [31,50,54] and pseudo-second-order [31,50,54] are given in Equations (4) and (5), respectively:

(4) $$\ln\;(q_{e}-q_{t})\;=\;\ln\;q_{e}\;+\;k_{1}t$$

(5) $$\frac{t}{q_{t}}=\;\frac{1}{k_{2}\;{q_{e}}^{2}}\;+\;\frac{1}{q_{e}}\cdot t$$

where *q_t_* and *q_e_* (mg·g^−1^) are the adsorption capacity of adsorbate at contact time *t* (min) and equilibrium time, respectively, *k*_1_ (min^−1^) and *k*_2_ (mg·g^−1^·min^−1^) are the adsorption rate constants of the pseudo-first-order and pseudo-second-order equation.

Table 7 represents the correlative parameters of adsorption kinetics of the adsorbate. Application of pseudo-first-order and pseudo-second-order models for the kinetic data of salicylic acid and mefenamic acid were presented in Figures S3 and S4, respectively. It was noticeable that pseudo-second-order model is suitable to describe the adsorption kinetic data since the theoretical *q_e_* values are very close to the experimental *q_e_* values, with the correlation coefficient (R^2^) greater than 0.99, implying the best fitting model for the kinetic data. The pseudo-second-order suggests that the adsorption follows second order chemisorption [55]. It can be concluded that the adsorption of salicylic acid and mefenamic acid by HXL poly(AN-*co*-EGDMA-*co*-VBC) was mainly by chemisorption.

### 3.4. Adsorption Mechanism

**Figure 10:**
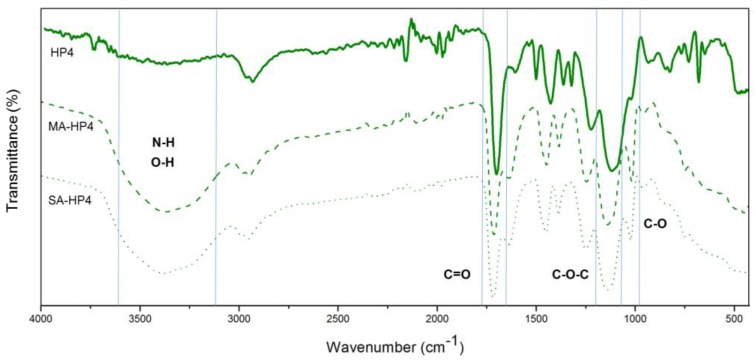
FT-IR spectra of HXL poly (AN-*co*-EGDMA-*co*-VBC) (HP4) after mefenamic acid (MA-HP4) and salicylic acid (SA-HP4) adsorption onto HP4.

The mechanism of adsorption were interpreted by comparing the FT-IR spectra of adsorbent-before adsorption (HP4) with adsorbent-after adsorption (SA-HP4 and MA-HP4). As shown in Figure 10, the absorption bands at 3382 cm^−1^ and 3369 cm^−1^, that were assigned to the O–H group of salicylic acid and mefenamic acid, were observed in both SA-HP4 and MA-HP4 spectra. In addition, from the spectrum of MA-HP4, the band identified at ~3370 cm^−1^ is due to the N–H stretching vibration of mefenamic acid. The appearance of C–O of hydroxyl group stretching vibration was observed at 1023 cm^−1^ and 1021 cm^−1^ in the spectra of SA-HP4 and MA-HP4, respectively. The strong vibration at 1720 cm^−1^ corresponds to the C=O stretch of EGDMA was shifted to 1728 cm^−1^ (SA-HP4) and 1731 cm^−1^ (MA-HP4). The band that was assigned to the C–O–C stretch was shifted to 1150 cm^−1^ and 1151 cm^−1^ after the adsorption. The shifting of the absorption bands might be due to the formation of new bonds between HXL poly(AN-*co*-EGDMA-*co*-VBC) with salicylic acid and mefenamic acid. Thus, this can be proved that salicylic acid and mefenamic acid have been adsorbed onto HXL poly(AN-*co*-EGDMA-*co*-VBC).

### 3.5. Proposed Mechanism of Salicylic Acid and Mefenamic Acid Adsorption by HXL Poly(AN-co-EGDMA-co-VBC)

**Figure 11:**
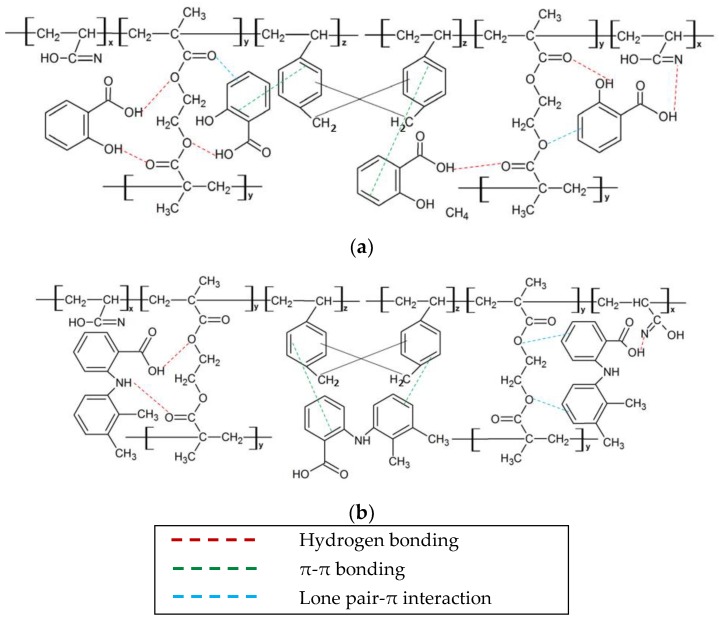
Proposed mechanism of adsorption of (**a**) salicylic acid and (**b**) mefenamic acid by HXL poly (AN-*co*-EGDMA-*co*-VBC).

Figure 11 represents the proposed mechanism of adsorption of pharmaceuticals by HXL poly (AN-*co*-EGDMA-*co*-VBC). Since the specific surface area of the hypercrosslinked polymer is quite low (59.1 m^2^·g^−1^), the adsorption might have been majorly contributed by the interaction between active sites on the surface of the hypercrosslinked polymer with the pharmaceuticals. Adsorption of pharmaceuticals was contributed by hydrogen bonding, π–π bonding and lone pair–π interaction. A hydrogen bond is formed by the attraction of hydrogen atom of mefenamic and salicylic acid molecules to the oxygen atom of EGDMA molecules. In addition, the attraction of the nitrogen atom of imine group derived from the nitrile group of acrylonitrile comonomer to the hydrogen atom of salicylic and mefenamic acid molecules formed hydrogen bonding. In addition to that, the benzene ring of salicylic and mefenamic acid molecules is attracted to the benzene ring of vinylbenzyl chloride comonomer and this formed π–π bonding. The interaction between lone pair of the oxygen atom of EGDMA molecule and benzene ring of pharmaceuticals molecules formed lone pair–π interaction. For salicylic acid, lone pair–π interaction was also formed by the interaction of the lone pair of an oxygen atom from the carbonyl group of EGDMA molecule and the benzene ring of salicylic acid molecule.

## 4. Conclusions

Poly(AN-*co*-EGDMA-*co*-VBC) was successfully synthesized by precipitation polymerization with 39%–48% yields of polymerization. Hypercrosslinking of poly(AN-*co*-EGDMA-*co*-VBC) via a Friedel–Crafts reaction showed an increase in specific surface area which is up to 30%. The low increment of the surface area might due to the low amount of VBC used and the absence of stabilizer which helps in producing polymers with well-defined spherical particles along with a high number of pores. These polymers with high polarity arising from the inclusion of AN and EGDMA monomers were utilized as sorbents to capture polar pharmaceuticals from solution. Adsorption kinetic data obeyed pseudo-second-order rate equation. The maximum adsorption capacity of salicylic acid and mefenamic acid measured by Langmuir model were up to 416.7 and 625 mg·g^−1^, respectively. The equilibrium adsorption data of salicylic acid were best described by the Freundlich model. Meanwhile, the equilibrium adsorption data of mefenamic acid were well fitted with Langmuir model. For future recommendation, hypercrosslinked terpolymer with a high amount of VBC will be synthesized by suspension polymerization with the aid of stabilizer to further increase the specific surface area as well as to form a polymer with monodisperse spherical beads.

## Figures and Tables

**Table 1:** Monomers in feed for synthesis of polymers.

Sample	AN (mol%)	EGDMA (mol%)	VBC (mol%)	AN (mL)	EGDMA (mL)	VBC (mL)
P1	100	-	-	4.938	-	-
P4	25	70	5	0.410	3.308	0.177
P7	20	75	5	0.314	3.390	0.169

**Table 2:** Yields of poly(AN) and poly(AN-*co*-EGDMA-*co*-VBC) in a mixture of acetonitrile/toluene.

Sample	AN/EGDMA/VBC (mol%)	Yields (%)
P1	100/0/0	3
P4	25/70/5	39
P7	20/75/5	48

**Table 3:** Elemental microanalysis data of poly(AN) and poly(AN-*co*-EGDMA-*co*-VBC) before and after hypercrosslinking reactions.

		Elemental Microanalysis (%)				
Sample	AN/EGDMA/VBC (mol%)	C	H	N	O	Cl
P1	100/0/0	63.08	5.49	25.39	-	-
P4	25/70/5	57.43	6.63	0.51	27.40	7.55
HP4	25/70/5	54.13	6.25	1.17	32.55	5.48
P7	20/75/5	57.70	6.70	0.40	29.81	4.95
HP7	20/75/5	52.12	6.16	0.17	29.30	11.58

**Table 4:** Textural parameters of poly(AN) and poly(AN-*co*-EGDMA-*co*-VBC) before and after hypercrosslinking reactions.

Sample	AN/EGDMA/VBC (mol%)	Specific Surface Area (m^2^·g^−1^)	Specific Pore Volume (cm^3^·g^−1^)	Mean Pore Size (nm)
P1	100/0/0	2	-	-
P4	25/70/5	45.6	0.851	74.7
HP4	25/70/5	59.1	0.631	42.7
P7	20/75/5	47.4	0.350	29.5
HP7	20/75/5	1.1	0.118	447.0

**Table 5:** Langmuir and Freundlich isotherm parameters.

	Langmuir Model			Freundlich Model		
	*K_L_*	*q_m_*	R^2^	*K_F_*	n	R^2^
Salicylic acid						
HP4	4.420 × 10^−2^	416.7	0.9275	66.188	2.9709	0.9717
P4	2.555 × 10^−2^	333.3	0.7854	32.779	2.4096	0.8582
P1	3.473 × 10^−2^	149.2	0.7532	15.393	2.2568	0.8005
Mefenamic acid						
HP4	1.673 × 10^−3^	625.0	0.9950	2.3085	0.8061	0.9955
P4	1.741 × 10^−3^	370.4	0.9762	4.5172	0.7765	0.9698
P1	4.319 × 10^−3^	84.7	0.9120	6.3777	0.7567	0.8967

**Table 6:** Comparison of maximum adsorption capacity of salicylic acid and mefenamic acid with those reported adsorbents.

Adsorbents	BET Surface Area (m^2^·g^−1^)	*q_max_* (mg·g^−1^)	References
**Salicylic acid**			
Magnetic molecularly imprinted polymer	-	36.8	[6]
Magnetic non-imprinted polymer	-	6.5	[6]
Hypercrosslinked DVB/VBC	1027	333.3	[15]
Methoxy modified hypercrosslinked DVB/VBC	848	492.6	[15]
Phenoxy modified hypercrosslinked DVB/VBC	875	621.1	[15]
Hypercrosslinked poly(VBC-*co*-DVB-*co*-MA)	1047	584.3	[19]
Modified hypercrosslinked poly(VBC-*co*-DVB-*co*-MA)	1010	457.9	[19]
HP4	59.1	416.7	This work
P4	45.6	333.3	This work
**Mefenamic acid**			
Clay micelle complex	-	100.0	[7]
Activated charcoal	-	90.9	[7]
Poly allyl glycidyl ether /iminodiacetic acid-co-N,N-dimethylacrylamide grafted to silica gel	-	7.0	[8]
Poly urea formaldehyde-bentonite	-	28	[52]
HP4	59.1	625.0	This work
P4	45.6	370.4	This work

**Table 7:** The correlative parameters of adsorption kinetics of salicylic acid and mefenamic acid.

	*q_e_* (exp)	Pseudo-First-Order			Pseudo-Second-Order		
		*k*_1_	*q_e_*	R^2^	*k*_2_	*q_e_*	R^2^
Salicylic acid							
HP4	169.5	0.1422	41.1	0.6651	1.934 × 10^−2^	169.5	0.9996
P4	144.1	0.1739	39.9	0.7106	1.536 × 10^−2^	144.9	0.9988
P1	61.9	0.1139	34.8	0.6895	4.956 × 10^−3^	66.7	0.9329
Mefenamic acid							
HP4	73.1	0.1292	27.5	0.7410	2.048 × 10^−2^	72.5	0.9970
P4	52.6	0.1423	15.3	0.7069	4.395 × 10^−2^	52.4	0.9988
P1	16.9	0.1396	11.5	0.8052	1.985 × 10^−2^	18.3	0.9652

## Supplementary Materials

The following are available online at https://www.mdpi.com/2073-4360/12/2/423/s1. Figure S1: Langmuir adsorption isotherms for the adsorption of (a) salicylic acid and (b) mefenamic acid onto HXL poly(AN-co-EGDMA-co-VBC) (HP4), poly(AN-co-EGDMA-co-VBC) (P4), and poly(AN) (P1), Figure S2: Freundlich adsorption isotherms for the adsorption of (a) salicylic acid and (b) mefenamic acid onto HXL poly(AN-co-EGDMA-co-VBC) (HP4), poly(AN-co-EGDMA-co-VBC) (P4), and poly(AN) (P1), Figure S3: Application of pseudo-first-order model for the kinetic data of (a) salicylic acid and (b) mefenamic acid, Figure S4: Application of pseudo-second-order model for the kinetic data of (a) salicylic acid and (b) mefenamic acid.

## References

[1] Ensano B., Borea L., Naddeo V., Belgiorno V., Luna M.D., Ballesteros F. (2017). Removal of Pharmaceuticals from Wastewater by Intermittent Electrocoagulation. Water.

[2] Wilkinson J., Boxall A., Kolpin D. (2019). A Novel Method to Characterise Levels of Pharmaceutical Pollution in Large-Scale Aquatic Monitoring Campaigns. Appl. Sci..

[3] Phonsiri V., Choi S., Nguyen C., Tsai Y.-L., Coss R., Kurwadkar S. (2019). Monitoring occurrence and removal of selected pharmaceuticals in two different wastewater treatment plants. SN Appl. Sci..

[4] Carbone C., Musumeci T., Pignatello R. (2013). Non-steroidal anti-inflammatory drugs. Drug Biomembr. Interact. Stud..

[5] Al-Odaini N.A., Zakaria M.P., Yaziz M.I., Surif S., Abdulghani M. (2013). The occurrence of human pharmaceuticals in wastewater effluents and surface water of Langat River and its tributaries, Malaysia. Int. J. Environ. Anal. Chem..

[6] Zhang Z., Niu D., Li Y., Shi J. (2018). Magnetic, core-shell structured and surface molecularly imprinted polymers for the rapid and selective recognition of salicylic acid from aqueous solutions. Appl. Surf. Sci..

[7] Khalaf S., Al-Rimawi F., Khamis M., Nir S., Bufo S.A., Scrano L., Karaman R. (2015). Efficiency of Membrane Technology Activated Charcoal and a Clay Micelle Complex for the Removal of Ibuprofen and Mefenamic Acid. Int. Case Stud. J..

[8] Sadeghi H.B., Panahi H.A., Mahabadi M., Moniri E. (2015). Preconcentration and Determination of Mefenamic Acid in Pharmaceutical and Biological Fluid Samples by Polymer-grafted Silica Gel Solid-phase Extraction Following High Performance Liquid Chromatography. Iran. J. Pharm. Res..

[9] Zhou F., Man R., Huang J. (2019). Hyper-cross-linked polymers functionalized with primary amine and its efficient adsorption of salicylic acid from aqueous solution. J. Chem. Thermodyn..

[10] Gan Y., Chen G., Sang Y., Zhou F., Man R., Huang J. (2019). Oxygen-rich hyper-cross-linked polymers with hierarchical porosity for aniline adsorption. Chem. Eng. J..

[11] Wang X., Ou H., Huang J. (2019). One-pot synthesis of hyper-cross-linked polymers chemically modified with pyrrole, furan, and thiophene for phenol adsorption from aqueous solution. J. Colloid Interface Sci..

[12] Wang X., He J., Huang J. (2019). Amino-modified hyper-cross-linked polymers with hierarchical porosity for adsorption of salicylic acid from aqueous solution. J. Chem. Thermodyn..

[13] Wang X., Mao X., Huang J. (2018). Hierarchical porous hyper-cross-linked polymers modified with phenolic hydroxyl groups and their efficient adsorption of aniline from aqueous solution. Colloids Surf. A Physicochem. Eng. Asp..

[14] Subri N.N.S., Cormack P.A.G., Jamil S.N.A.M., Abdullah L.C., Daik R. (2018). Synthesis of poly(acrylonitrile-*co*-divinylbenzene-*co*-vinylbenzyl chloride)-derived hypercrosslinked polymer microspheres and a preliminary evaluation of their potential for the solid-phase capture of pharmaceuticals. J. Appl. Polym. Sci..

[15] Zhou F., Man R., Huang J. (2018). Alkoxy-Modified Hyper-Cross-Linked Polymers with Hierarchical Porosity and Their Adsorption of Salicylic Acid from Aqueous Solution. Ind. Eng. Chem. Res..

[16] Zhang T., Zhou F., Huang J., Man R. (2018). Ethylene glycol dimethacrylate modified hyper-cross-linked resins: Porogen effect on pore structure and adsorption performance. Chem. Eng. J..

[17] Zhang T., Huang J. (2017). Tunable synthesis of the polar modified hyper-cross-linked resins and application to the adsorption. J. Colloid Interface Sci..

[18] Wang X., Zhang T., Huo J., Huang J., Liu Y.-N. (2017). Tunable porosity and polarity of polar post-cross-linked resins and selective adsorption. J. Colloid Interface Sci..

[19] Fu Z., Huang J. (2017). Polar hyper-cross-linked resin with abundant micropores/mesopores and its enhanced adsorption toward salicylic acid: Equilibrium, kinetics, and dynamic operation. Fluid Phase Equilibria.

[20] Xiao G., Wen R., Liu A., He G., Wu D. (2017). Adsorption performance of salicylic acid on a novel resin with distinctive double pore structure. J. Hazard. Mater..

[21] Shao L., Li Y., Zhang T., Liu M., Huang J. (2017). Controllable Synthesis of Polar Modified Hyper-Cross-Linked Resins and Their Adsorption of 2-Naphthol and 4-Hydroxybenzoic Acid from Aqueous Solution. Ind. Eng. Chem. Res..

[22] Urban J., Svec F., Fréchet Jean M.J. (2010). Efficient Separation of Small Molecules Using a Large Surface Area Hypercrosslinked Monolithic Polymer Capillary Column. Anal. Chem..

[23] Yu W., Sun W., Xu C., Wang C., Jia Y., Qin X., Xie C., Yu S., Xian M. (2018). Effective adsorption toward p-aminobenzoic acid from aqueous solution by a L-malic acid modified hyper-crosslinked resin: Equilibria and kinetics. J. Taiwan Inst. Chem. Eng..

[24] Yahya M.Z., Abdullah N. (2017). Preparation and Characterization of Hypercrosslinked Poly (HEMA-*co*-EGDMA-*co*-VBC). Indian J. Sci. Technol..

[25] Tan L., Tan B. (2017). Hypercrosslinked porous polymer materials: Design, synthesis, and applications. Chem. Soc. Rev..

[26] Fontanals N., Cormack P.A., Sherrington D.C. (2008). Hypercrosslinked polymer microspheres with weak anion-exchange character. J. Chromatogr. A.

[27] Fontanals N., Cortés J., Galià M., Marcé R.M., Cormack P.A.G., Borrull F., Sherrington D.C. (2005). Synthesis of Davankov-type hypercrosslinked resins using different isomer compositions of vinylbenzyl chloride monomer, and application in the solid-phase extraction of polar compounds. J. Polym. Sci. Part A Polym. Chem..

[28] Fontanals N., Galià M., Cormack P.A., Marcé R.M., Sherrington D.C., Borrull F. (2005). Evaluation of a new hypercrosslinked polymer as a sorbent for solid-phase extraction of polar compounds. J. Chromatogr. A.

[29] Tsyurupa M., Davankov V. (2006). Porous structure of hypercrosslinked polystyrene: State-of-the-art mini-review. React. Funct. Polym..

[30] Fontanals N., Marcé R., Borrull F. (2005). New hydrophilic materials for solid-phase extraction. TrAC Trends Anal. Chem..

[31] Galicia-Aguilar J.A., Santamaría-Juárez J.D., López-Badillo M., Sánchez-Cantú M., Varela-Caselis J.L. (2017). Synthesis and characterization of AN/EGDMA-based adsorbents for phenol adsorption. React. Funct. Polym..

[32] Jose J., John M., Mathew B. (2003). Effect of the Nature of Crosslinking Agent on the Catalase-Like Activity of Polystyrene-Bound Glycine–Metal Complexes. J. Macromol. Sci. Part A.

[33] Huang J., Zhu J., Snyder S.A., Morris A.J., Turner S.R. (2018). Nanoporous highly crosslinked polymer networks with covalently bonded amines for CO_2_ capture. Polymer.

[34] Liu W., Zhu X., Yang X., Li K., Yang Z. (2018). Preparation of highly cross-linked hydrophilic porous microspheres poly(N,N′-methylenebisacrylamide) and poly(N,N′-methylenebisacrylamide-*co*-acrylic acid) with an application on the removal of cadmium. Polym. Adv. Technol..

[35] El-Bahy S.M., El-Bahy Z.M. (2017). Immobilization of 2-amino pyridine onto poly(acrylonitrile-*co*-N,N′-methylenebisacrylamide) nanoparticles for the removal of Hg(II), Cd(II) and Cr(III): Batch and column techniques. J. Environ. Chem. Eng..

[36] El-Bahy S.M., El-Bahy Z.M. (2016). Synthesis and characterization of polyamidoxime chelating resin for adsorption of Cu(II), Mn(II) and Ni(II) by batch and column study. J. Environ. Chem. Eng..

[37] Kulygin O., Silverstein M.S. (2007). Porous poly(2-hydroxyethyl methacrylate) hydrogels synthesized within high internal phase emulsions. Soft Matter.

[38] Gokmen M.T., Prez F.E.D. (2012). Porous polymer particles—A comprehensive guide to synthesis, characterization, functionalization and applications. Prog. Polym. Sci..

[39] Özer E.T., Osman B., Kara A., Beşirli N., Gücer Ş., Sözeri H. (2012). Removal of diethyl phthalate from aqueous phase using magnetic poly(EGDMA–VP) beads. J. Hazard. Mater..

[40] Bagheri B., Abdouss M., Aslzadeh M.M., Shoushtari A.M. (2010). Efficient Removal of Cr^3+^, Pb^2+^ and Hg^2+^ Ions from Industrial Effluents by Hydrolyzed/Thioamidated Polyacrylonitrile Fibres. Iran. Polym. J..

[41] Hwang K.S., Choi W.J., Kim J.-H., Lee J.-Y. (2015). Preparation of hypercrosslinked poly(DVB-VBC) particles with high surface area and structured meso- and micropores. Macromol. Res..

[42] Wang R., Zhang Y., Ma G., Su Z. (2006). Preparation of uniform poly(glycidyl methacrylate) porous microspheres by membrane emulsification–polymerization technology. J. Appl. Polym. Sci..

[43] Li W.-H., Stöver H.D.H. (2000). Monodisperse Cross-Linked Core-Shell Polymer Microspheres by Precipitation Polymerization. Macromolecules.

[44] Norhayati A., Zuhaili Y.M., Rabiatuladawiah M. (2018). Synthesis and characterization of poly(HEMA-*co*-EGDMA-*co*-VBC) by modified suspension polymerization: Effects of polymerization parameters reaction on chemical and thermal properties of polymer. Mater. Today Proc..

[45] Yuan H.G., Kalfas G., Ray W.H. (1991). Suspension Polymerization. J. Macromol. Sci. Part C Polym. Rev..

[46] Liu F., Chen S., Gao Y. (2017). Synthesis of porous polymer-based solid amine adsorbent: Effect of pore size and amine loading on CO_2_ adsorption. J. Colloid Interface Sci..

[47] Adeyi A.A., Jamil S.N.A.M., Abdullah L.C., Choong T.S.Y., Lau K.L., Abdullah M. (2019). Adsorptive Removal of Methylene Blue from Aquatic Environments Using Thiourea-Modified Poly(Acrylonitrile-*co*-Acrylic Acid). Materials.

[48] Adeyi A.A., Jamil S.N.A.M., Abdullah L.C., Choong T.S.Y. (2019). Adsorption of Malachite Green Dye from Liquid Phase Using Hydrophilic Thiourea-Modified Poly(acrylonitrile-*co*-acrylic acid): Kinetic and Isotherm Studies. J. Chem..

[49] Leshchinskaya A., Ezhova N., Pisarev O. (2016). Synthesis and characterization of 2-hydroxyethyl methacrylate-ethylene glycol dimethacrylate polymeric granules intended for selective removal of uric acid. React. Funct. Polym..

[50] Boudouaia N., Bengharez Z., Jellali S. (2019). Preparation and characterization of chitosan extracted from shrimp shells waste and chitosan film: Application for Eriochrome black T removal from aqueous solutions. Appl. Water Sci..

[51] Haghseresht F., Lu G.Q. (1998). Adsorption Characteristics of Phenolic Compounds onto Coal-Reject-Derived Adsorbents. Energy Fuels.

[52] Majeed B.A.A., Muhseen R.J., Jassim N.J. (2017). Adsorption of Mefenamic Acid from Water by Bentonite Poly urea formaldehyde Composite Adsorbent. J. Eng..

[53] Fallou H., Cimetière N., Giraudet S., Wolbert D., Cloirec P.L. (2016). Adsorption of pharmaceuticals onto activated carbon fiber cloths—Modeling and extrapolation of adsorption isotherms at very low concentrations. J. Environ. Manag..

[54] Varga M., Elabadsa M., Tatár E., Mihucz V.G. (2019). Removal of selected pharmaceuticals from aqueous matrices with activated carbon under batch conditions. Microchem. J..

[55] Jayalakshmi R., Jeyanthi J. (2019). Simultaneous removal of binary dye from textile effluent using cobalt ferrite-alginate nanocomposite: Performance and mechanism. Microchem. J..

