# Supplementary material for: Preparation of Ethylene Glycol Dimethacrylate (EGDMA)-Based Terpolymer as Potential Sorbents for Pharmaceuticals Adsorption
Source: Polymers (Basel). 2020 Feb 12;12(2):423. doi: 10.3390/polym12020423 (PMC7077665; doi:10.3390/polym12020423)
Supplement: Supplementary file 1 [file polymers-12-00423-s001.pdf]

***Supplementary Materials***

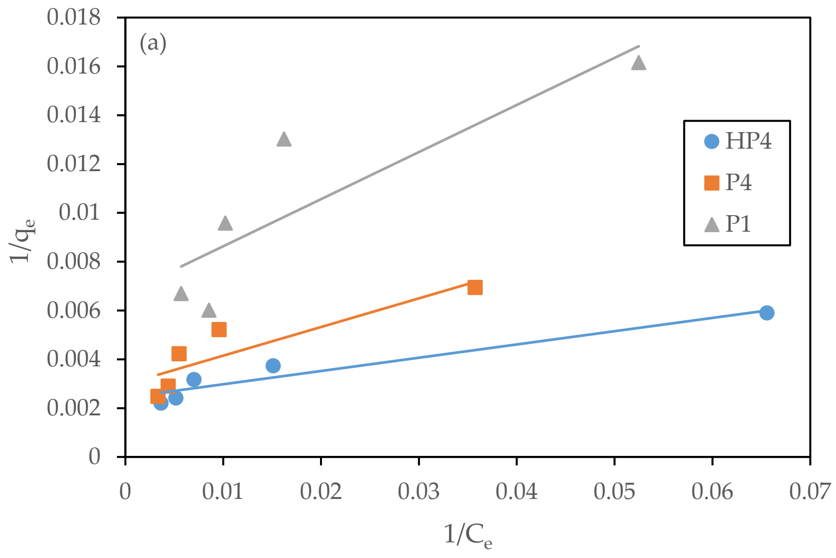


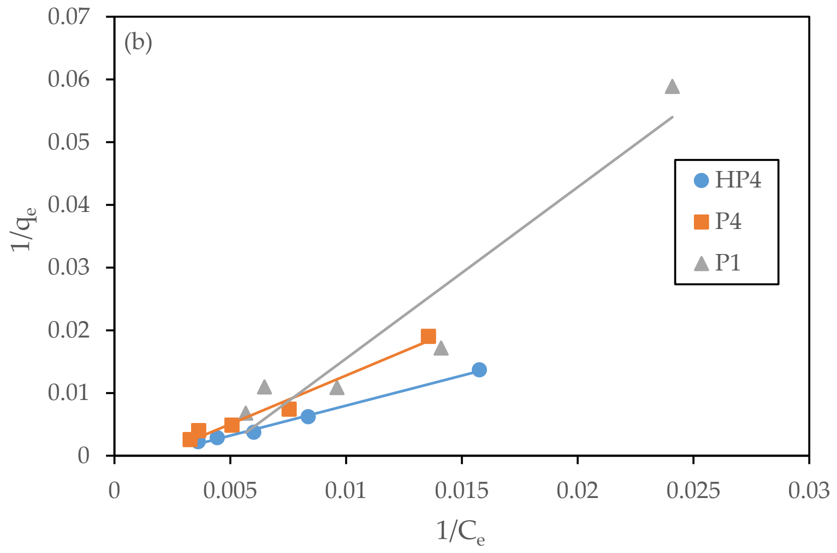


**Figure S1** Langmuir adsorption isotherms for the adsorption of (a) salicylic acid and (b) mefenamic acid onto HXL poly(AN-*co*-EGDMA-*co*-VBC) (HP4), poly(AN-*co*-EGDMA-*co*-VBC) (P4), and poly(AN) (P1).

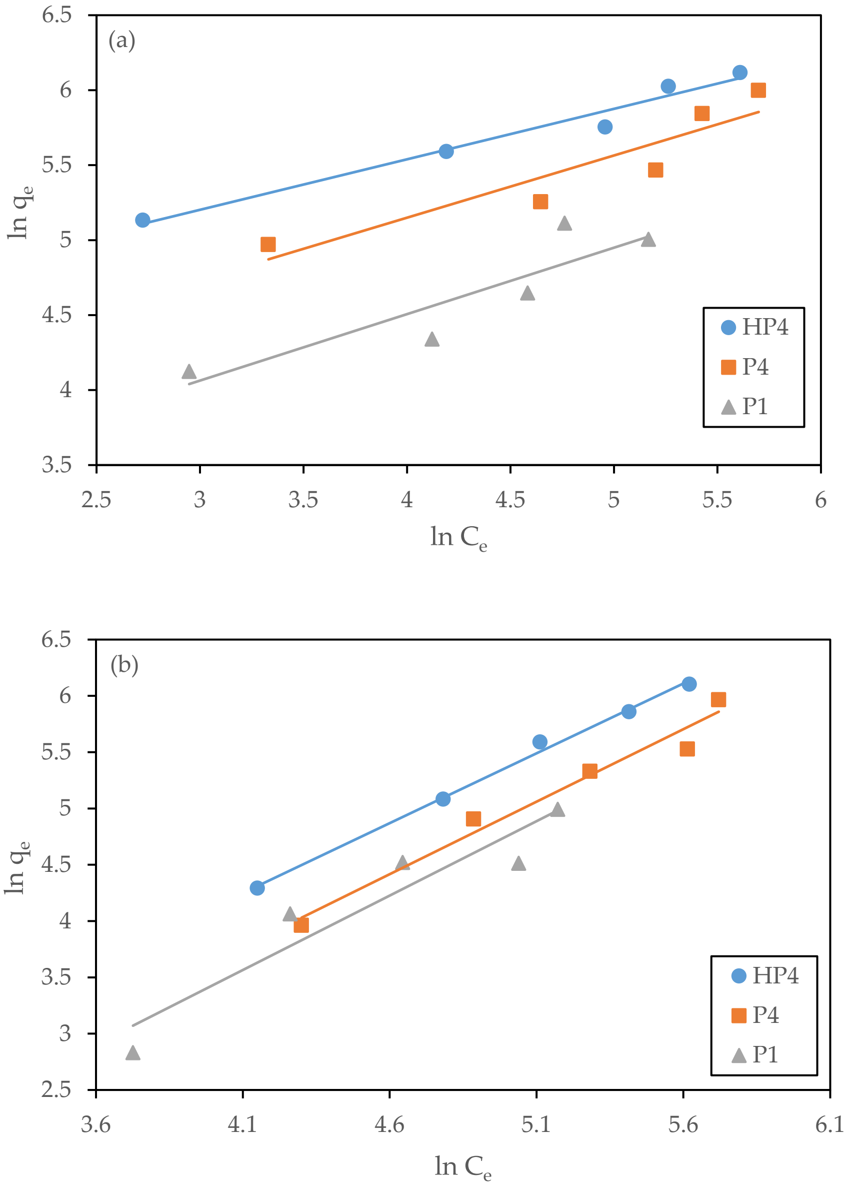


**Figure S2** Freundlich adsorption isotherms for the adsorption of (a) salicylic acid and (b) mefenamic acid onto HXL poly(AN-*co*-EGDMA-*co*-VBC) (HP4), poly(AN-*co*-EGDMA-*co*-VBC) (P4), and poly(AN) (P1).

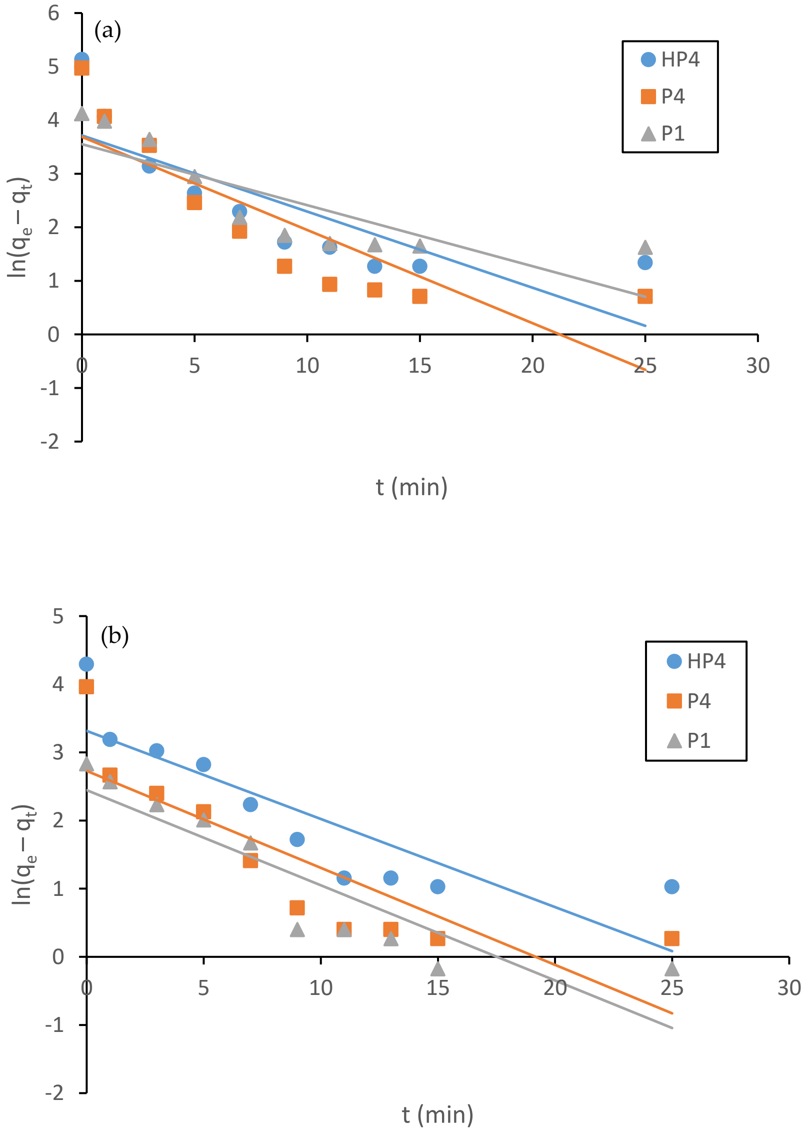


**Figure S3** Application of pseudo-first-order model for the kinetic data of (a) salicylic acid and (b) mefenamic acid.

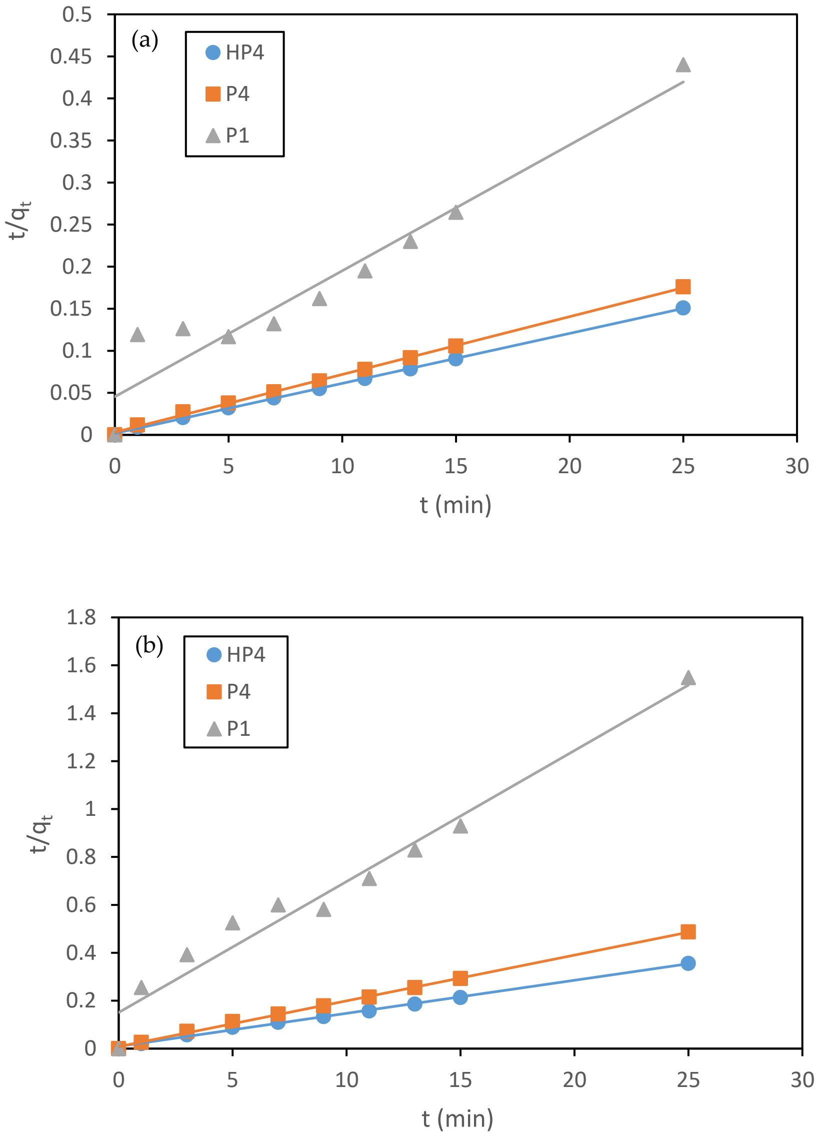


**Figure S4** Application of pseudo-second-order model for the kinetic data of (a) salicylic acid and (b) mefenamic acid.
